# Management strategies for burning mouth syndrome: a comprehensive review

**DOI:** 10.22514/jofph.2026.001

**Published:** 2026-01-12

**Authors:** Federica Canfora, Noemi Coppola, Elena Calabria, Niccolò Giuseppe Armogida, Michele Davide Mignogna, Gianrico Spagnuolo, Daniela Adamo

**Affiliations:** ^1^Department of Neurosciences, Reproductive Sciences and Odontostomatology, University of Naples “Federico II”, 80131 Naples, Italy; ^2^Department of Health Sciences, School of Dentistry, University Magna Graecia of Catanzaro, 88100 Catanzaro, Italy; ^3^Therapeutic Dentistry Department, Institute for Dentistry, Sechenov University, 119991 Moscow, Russia; ^4^Department of Life Science, Health, and Health Professions, Link Campus University, 00165 Rome, Italy

**Keywords:** Burning mouth syndrome, Dysesthetic sensation, Full functional recovery, Vortioxetine, Clonazepam

## Abstract

Burning Mouth Syndrome (BMS) is a complex chronic neuropathic orofacial pain disorder characterized by a persistent burning or dysesthetic sensation in the oral cavity without an identifiable organic cause. The management of BMS has evolved beyond symptom relief to focus on achieving full functional recovery (FFR), which encompasses restoring patients to their usual activities without restrictions, addressing both physical and psychological dimensions. Key pharmacological treatments such as clonazepam and capsaicin are explored in detail, alongside the potential of newer agents like various classes of antidepressants (including tricyclic antidepressants, selective serotonin reuptake inhibitors, and serotonin and noradrenaline reuptake inhibitors, vortioxetine) and antiepileptics showing promise in addressing the multifactorial nature of BMS. Non-pharmacological interventions, such as cognitive-behavioral therapy (CBT), low-level laser therapy (LLLT), and transcranial magnetic stimulation (TMS), are highlighted for their potential to complement pharmacological treatments. These interventions aim to modify pain perception, reduce psychological burdens, and enhance overall quality of life. Lifestyle modifications, including dietary changes, stress management techniques, improved sleep hygiene, and regular physical activity, are essential components of a holistic treatment plan that addresses modifiable risk factors affecting brain health. The integration of telemedicine and digital health resources is proposed to enhance patient management and accessibility to multidisciplinary care. This review provides a comprehensive update on all available therapeutic approaches for BMS, encompassing pharmacological treatments, non-pharmacotherapeutic interventions, and lifestyle optimization strategies, offering a holistic perspective on managing this condition.

## 1. Introduction

Burning Mouth Syndrome (BMS) is defined according to the International Classification of Orofacial Pain (ICOP, 2020) [1] as an intraoral burning or dysaesthetic sensation, recurring daily for more than 2 hours per day for more than 3 months, in the absence of evident causative lesions on clinical examination and investigation. Epidemiological data indicate a global prevalence of approximately 1.73% in the general population, increasing to 7.72% among dental patients, as reported in the meta-analysis by Wu *et al*. [2]. The underlying mechanisms of BMS remain largely unclear, though current research supports the involvement of both central and peripheral nervous system dysfunctions. Endocrine imbalances and psychological factors may also contribute to its onset and persistence. Common comorbidities include mood disturbances, sleep disorders, and cognitive dysfunctions, which should be addressed as part of comprehensive patient care. Given its multifactorial etiology, BMS requires a tailored therapeutic approach that combines pharmacological treatments with non-pharmacological and lifestyle-based strategies. This review presents a current synthesis of available therapeutic options, highlighting the need for a multidisciplinary, patient-centered model to optimize clinical outcomes and enhance the quality of life in affected individuals.

BMS poses a therapeutic challenge that extends well beyond the mere reduction of pain. Contemporary care should aim for complete functional restoration—returning the patient to unrestricted social, occupational, and leisure activities—rather than simple symptom palliation. To reach this goal, treatment should concurrently address both peripheral and central mechanisms, in line with the recognition of BMS as a nociplastic pain condition. This approach also involves targeting modifiable contributors, such as anxiety, depression, sleep disturbances, and cognitive dysfunction [3]. Pharmacological agents (*e.g.*, neuromodulators, antidepressants) should be combined with an individualized package of non-drug measures. Core elements include structured patient education on disease mechanisms, correct medication use, dietary triggers, sleep hygiene, and stress management techniques. Practical self-care, avoiding spicy or acidic foods, selecting non-irritating oral products, sipping cold liquids, or chewing sugar-free gum can lessen burning sensations and stimulate saliva.

Psychological interventions, particularly cognitive-behavioural therapy (CBT) with mindfulness, relaxation training, and cognitive restructuring, help reframe pain perception and bolster emotional resilience. Where cognitive deficits are documented, cognitive rehabilitation or nootropic agents may be considered. Because the disorder is multifactorial, coordinated input from orofacial pain specialists, psychiatrists, neurologists, geriatricians, nutritionists, and psychologists is essential.

Treatment duration remains uncertain for chronic pain per se, but guidance from mood-disorder management suggests maintaining therapy for roughly 9–12 months after full functional recovery is reached [4] (Fig. 1). In essence, while Full Functional Recovery (FFR) represents the ultimate therapeutic goal, it remains challenging to achieve in chronic pain conditions. Pursuing FFR necessitates the comprehensive treatment of both central and peripheral neuropathy, as well as the management of modifiable risk factors that can impact brain health [3, 5] (Fig. 2).

**Fig. 1.** Comprehensive treatment approach for burning mouth syndrome (BMS). Aiming at Full Functional Recovery (FFR). Full functional recovery is defined as a return to normal functioning at home and work, along with regaining enjoyment in usual activities and relationships. The treatment plan encompasses multiple facets, including pain relief, management of mood disorders, stress reduction, and improvement of sleep quality. It also highlights the importance of cognitive performance enhancement and psychoeducation, ensuring patients are well-informed about their condition and treatment. Guidance on adopting healthy lifestyle behaviors and addressing modifiable risk factors is also crucial.

**Fig. 2.** Therapeutic outcomes in BMS. To achieve full functional recovery (FFR), treatment must address both peripheral and central neuropathy. This includes managing oral symptoms, improving sleep quality, treating mood disorders and cognitive impairment, and addressing modifiable risk factors, such as smoking, alcohol consumption, physical activity, nutrition, vitamin deficiencies, and attitudes about pain. BMS: Burning Mouth Syndrome.

Ultimately, optimal outcomes are more likely when care is provided by a multidisciplinary team, including pain specialists, mental health professionals, neurologists, geriatricians, nutritionists, and psychologists. Although, the implementation of such an approach may be limited in routine clinical practice, particularly in primary care or general dental settings. Interventions should be reviewed and adjusted regularly, ensuring they remain responsive to the evolving clinical picture. Long-term improvements in quality of life and symptom control depend on such coordinated, person-centered care.

## 2. Evidence, adherence, and the central role of therapeutic alliance

A recent systematic review assessing pharmacological interventions for BMS through an analysis of randomized controlled trials concluded that evidence supporting interventions varies, yet some pharmacological approaches demonstrate improved symptomatic outcomes [6].

Nevertheless, the current body of evidence supporting the preference of one therapy over another is presently inadequate, with significant influence from both the clinical judgment of healthcare providers and the priorities and expectations of patients.

Furthermore, a limited proportion, approximately 30%, of patients with BMS with a long-standing disease have reported experiencing relief following therapeutic intervention [7]. This evidence implies a potential increase in treatment efficacy if initiated promptly, underlining the importance of early diagnosis and therapeutic intervention in managing BMS to enhance patient outcomes [8].

Regimens for BMS can be administered topically, systemically, or in combination. While an evolving body of evidence underscores the association between BMS and psychological factors, supporting the use of anxiolytics and antidepressants in some cases, it is important to note that these medications may also be used for their neuromodulatory properties and recognized indication in the management of neuropathic pain [7, 9, 10, 11]. Yet, searching for a universally efficacious treatment encounters obstacles, primarily due to the limited scope of extensive randomized trials and their participant populations (Table 1).

**Table 1.** Pharmacological treatment of BMS.

Treatment		Dosage	Mechanism of action	Efficacy	Considerations
Topical					
	Clonazepam	0.5 mg/mL solution, 1 mg disintegrating tablet 0.5–3 mg/day	GABA-A agonist, long-acting benzodiazepine	Significant reduction in pain (NRS)	Preferred for localized treatment with fewer systemic effects.
	Capsaicin	0.02% solution oral rinse 0.01/0.025% gel	TRPV1 agonist on nociceptive C fibers	Significant reduction in pain	May cause initial increase in burning sensation; evaluate adverse effects.
	Bupivacaine Lozenges	5% lozenge three times a day	Local anesthetic	Borderline significant reduction in pain	Short-term efficacy; potential benefit over placebo.
	Lidocaine gel	Typically 2%–4% concentration; applied topically up to 3–4 times daily	Blocks voltage-gated sodium channels in peripheral nerves, preventing pain signal transmission	Shown to increase sensory thresholds and provide temporary relief of burning pain in BMS patients	Short duration of action; generally well tolerated; avoid overuse to reduce risk of mucosal irritation or systemic absorption, especially in elderly patients.
	Gabapentin	250 mg/mL 5 mL of the solution swish and split 2–4 times a day	Structural analogue of GABA; binds to voltage-dependent calcium channels	Significant reduction in pain (NRS)	The drug was evaluated in a retrospective study. No serious adverse events were reported.
	Amitriptyline	2% solution, applied 2–4 times per day	Local sodium channel blockade	Significant reduction in pain intensity	Side effects such as dry mouth and bitter taste reported; can be minimized with bedtime application and lower concentrations.
Systemic					
	Clonazepam	0.5–1 mg daily	GABA-A agonist, long-acting benzodiazepine	Significant improvement in pain (VAS; NRS)	Risk of dependency; requires careful monitoring.
	Pregabalin	75–300 mg daily in divided doses	Binds to the α2δ subunit of voltage-gated calcium channels in the CNS, reducing excitatory neurotransmitter release and neuronal hyperexcitability; also modulates amygdala activity contributing to its anxiolytic effect	Demonstrated moderate to high efficacy in reducing burning sensations and neuropathic pain in BMS; effective in comorbid anxiety, enhancing overall symptom control; especially beneficial in combination with SSRIs/SNRIs or vortioxetine	Requires individualized titration to balance efficacy and tolerability; common side effects include dizziness, somnolence, and weight gain; may be preferred over gabapentin due to quicker onset and better tolerability in some studies.
	Gabapentin	300–2400 mg daily in divided doses	Structural analogue of GABA; binds to voltage-dependent calcium channels	Effective in reducing pain symptoms	Widely used for neuropathic pain; dose dependent on tolerance and response.
	Alpha-Lipoic Acid (ALA)	600–800 mg daily	Antioxidant and neuroprotective agent	Mixed results; some studies suggest benefit	ALA decreases oxidative damage in the nervous system. It has a broad spectrum of action towards many free radical species and boosts the endogenous antioxidant systems.
	Amitriptyline	5–150 mg daily at bedtime	Triciclic antidepressants (TCAs)	Demonstrated significant pain relief in BMS, especially effective in patients with comorbid anxiety or sleep disturbances	TCAs with analgesic properties; monitor for side effects (dry mouth, dizziness, blurred vision, constipation and urinary retention).
	Nortriptyline	10–30 mg daily at bedtime	Triciclic antidepressants (TCAs)	Moderate improvement in dpain symptoms	TCAs with analgesic properties; monitor for side effects dry mouth, dizziness, blurred vision, constipation and urinary retention.
	Duloxetine	20–60 mg daily	Serotonin and noradrenaline reuptake inhibitor (SNRI)	Effective in reducing neuropathic pain	SNRI used for modulating pain perception and improved mood. Careful monitoring of side effects (QTc prolongation, increase blood pression, increase prolactin level, sweating, dizziness).
	Venlafaxine	75–150 mg daily	Serotonin and noradrenaline reuptake inhibitor (SNRI)	Reduce BMS-related burning and improve associated anxiety and depressive symptoms	SNRI used for modulating pain perception and improved mood. Careful monitoring of side effects (QTc prolongation, increase blood pression, increase prolactin level, sweating, dizziness).
	Trazodone	200 mg daily 50–100 mg	Serotonin antagonist and reuptake inhibitors (SARIs)	Used for its sedative and anxiolytic effects	Exact efficacy in modulating pain is unclear. Low dosage to improve sleep. Side effects at higher dosage: dizziness and drowsiness, dry mouth.
	Paroxetine	20 mg daily	Selective serotonin reuptake inhibitor (SSRI)	Moderate improvement in pain symptoms and mood	SSRI used for modulating pain perception and improved mood. Careful monitoring of side effects (QTc prolongation, increase prolactin level, sexual disturbance, weight gain).
	Sertraline	50 mg daily	Selective serotonin reuptake inhibitor (SSRI)	Moderate improvement in pain symptoms and mood	SSRI used for modulating pain perception and improved mood. Careful monitoring of side effects (QTc prolongation, increase prolactin level, sexual disturbance, weight gain).
	Escitalopram	10 mg daily	Selective serotonin reuptake inhibitor (SSRI)	Moderate improvement in pain symptoms and mood	SSRI used for modulating pain perception and improved mood. Careful monitoring of side effects (QTc prolongation, increase prolactin level, sexual disturbance, weight gain).
	Citalopram	20 mg daily	Selective serotonin reuptake inhibitor (SSRI)	Moderate improvement in pain symptoms and mood	SSRI used for modulating pain perception and improved mood. Careful monitoring of side effects (QTc prolongation, increase prolactin level, sexual disturbance, weight gain).
	Fluoxetine	20–40 mg daily	Selective serotonin reuptake inhibitor (SSRI)	Moderate improvement in pain symptoms and mood	SSRI used for modulating pain perception and improved mood. Careful monitoring of side effects (QTc prolongation, increase prolactin level, sexual disturbance, weight gain).
	Vortioxetine	10–20 mg daily	Multimodal antidepressant: modulation of the serotonergic receptors Block of SERT inhibition of serotonin transporter	Improvement of pain, anxiety, depression, sleep, and cognition	Analgesic, sedative and anxiolytic properties with limited side effects.
	Melatonin	0.5 mg/12 mg	Hormon that interact with melatonin receptors (MT1 and MT2)	Regulates sleep wake cycles improving sleep	Antioxidant properties; research on its direct pain-relief efficacy needed.

*GABA-A: Gamma-Aminobutyric Acid type A; NRS: Numerical Rating Scale; TRPV1: Transient Receptor Potential Vanilloid type 1; VAS: Visual Analog Scale; CNS: Central Nervous System; BMS: Burning Mouth Syndrome; MT1: Melatonin Receptor type 1; MT2: Melatonin Receptor type 2; SERT: Serotonin Transporter; QTc: Corrected QT Interval.*

However, establishing a robust therapeutic alliance is paramount before any drug prescription to reduce any dropping out [12]. Comprehensive information must be disseminated to both the patient and their family members. This should cover an array of crucial aspects, including the rationale behind the selection of a particular treatment, the expected benefits, the latency of action, any potential for drug dependency, the projected duration of the treatment course, and the possible adverse events, which are more likely to occur during the initial phase of treatment [13].

Clinicians must engage in regular assessments of the patient’s progress, solidifying the understanding that successful treatment outcomes hinge on the precise adherence to the prescribed regimen—both in terms of dosage and timing. It is not uncommon for patients to prematurely cease medication upon feeling an improvement in symptoms; however, literature emphatically suggests that to prevent a recurrence of symptoms, the medication must be continued for an adequate time and at least until FFR is achieved [4].

Furthermore, an open dialogue should be maintained between patients and their healthcare providers to determine the most appropriate juncture for gradually phasing out the medication, ensuring that this decision is reached collaboratively and based on a thorough evaluation of the patient’s long-term wellness trajectory.

## 3. Pharmacological approaches

### 3.1 Clonazepam

Clonazepam continues to be a cornerstone treatment for BMS [14, 15]. As a benzodiazepine with anticonvulsant properties, clonazepam exerts its analgesic action by modulating gamma-aminobutyric acid (GABA) receptors as an agonist, specifically the GABA-A receptor complex, ubiquitously expressed throughout the central and peripheral nervous systems; thereby enhancing the efficacy of the pain-modulating pathways within both the peripheral and central nervous systems (CNS) [16]. Its role in decreasing neuronal excitability and modulating muscle tone is well-documented, and it is also implicated in the potentiation of descending pain modulation pathways [16]. This modulation is achieved by facilitating chloride channel opening, resulting in sustained hyperpolarization that mitigates neuronal hyperexcitability and forestalls depolarization and subsequent deafferentation neuronal firing [17] (Fig. 3).

**Fig. 3.** Mechanism of action of antiepileptic drugs. Pregabalin binds with high affinity to the alpha-2-delta subunit of voltage-gated calcium channels in the central nervous system. This subunit is an auxiliary component of these channels and modulates their function. By binding to the alpha-2-delta subunit, pregabalin reduces the influx of calcium ions (Ca^2+^) into neurons. This inhibition of calcium entry is particularly important in presynaptic excitatory neurons. The decreased calcium influx leads to a reduced release of excitatory neurotransmitters such as glutamate, norepinephrine, and substance P. These neurotransmitters are involved in the transmission of pain and other signals in the nervous system. By reducing the release of these excitatory neurotransmitters, pregabalin modulates synaptic transmission and neuronal excitability. Clonazepam binds to a specific site on the GABA-A receptor complex, which is distinct from the binding site of the endogenous neurotransmitter GABA (gamma-aminobutyric acid). This site is often referred to as the benzodiazepine binding site. Once bound, clonazepam acts as a positive allosteric modulator. This means it enhances the effect of GABA on the GABA-A receptor. The binding of clonazepam increases the receptor’s affinity for GABA, making it easier for GABA to activate the receptor, and when activated by GABA, it allows chloride ions (Cl^−^) to flow into the neuron. Clonazepam, by increasing GABA’s effect, enhances the opening of this chloride ion channel. The influx of chloride ions into the neuron results in hyperpolarization of the neuronal membrane. This makes it more difficult for the neuron to reach the threshold potential required for firing an action potential. As a result, neuronal excitability is reduced, leading to an overall inhibitory effect on neurotransmission. NMDAR: N-Methyl-D-Aspartate Receptor; AMPAR/KAR: α-amino-3-hydroxy-5-methyl-4-isoxazolepropionic acid receptor/Kainate Receptor; GAD: Glutamic Acid Decarboxylase.

Emergent data from an experimental animal study have identified the presence of GABA-A receptors within the tongue’s nerve fibers, positing a mechanistic basis for the localized analgesic effect of clonazepam in BMS [18].

Recent trials have substantiated the efficacy of clonazepam in ameliorating BMS symptoms, with variability in administration modalities, topical versus systemic, notwithstanding [19, 20]. However, the precise parameters for optimal administration and dosage remain to be fully elucidated. When administered systemically, this drug shows a rapid onset of action and high bioavailability—reaching 90% within one to four hours post-oral administration—and its extensive half-life ranging from 30 to 40 hours, contribute to its effectiveness in providing sustained pain relief [9].

Topical application of clonazepam within the oral cavity has been reported to offer more immediate analgesic benefits compared to systemic intake, with patients experiencing pain reduction within 10 minutes after dissolving a clonazepam tablet intraorally [21]. However, this method’s analgesic effect tends to subside within three to four hours. The topical route, favored for its simplicity and rapid yet transient efficacy, permits repeated dosing with a reduced risk of systemic side effects.

Gremeau-Richard *et al*. [9] conducted a double-blind, randomized study evaluating the efficacy of topical clonazepam in a cohort of 48 BMS patients. Under this protocol, subjects were administered a 1 mg clonazepam tablet or placebo buccally, retaining saliva at the site of pain for three minutes before expectoration. This regimen was repeated three times a day for 14 days, demonstrating a reduction in the intensity of pain measured with Numeric Rating Scale (NRS) scale [9].

In a longitudinal study extending over six months, the authors examined 66 BMS patients, comparing the analgesic effect of topical clonazepam with placebo [22]. The study reported a marked decrease in Visual Analogue Scale (VAS) scores among the clonazepam cohort, reinforcing the efficacy of topical administration. Complementary findings by De Castro *et al*. [23], involving 18 BMS patients treated with 10 mL of topical clonazepam (oral rinse solution 1 mg/10 mL) over a similar two-week period, further corroborated the symptomatic relief provided by the drug.

Moreover, investigations into the systemic administration of clonazepam have also indicated positive outcomes. Grushka *et al*. [24] found consistent pain relief with daily administration of 0.25 mg of systemic clonazepam, proposing an escalation in dosage for non-responders. Similarly, a double-blind, placebo-controlled study by Heckmann *et al*. [19] with a 0.5 mg daily dosage demonstrated a significant reduction in pain ratings.

Furthermore, Çinar *et al*. [25] conducted a comparative analysis of clonazepam, pregabalin, and alpha-lipoic acid (ALA) in three patient groups of 30 patients, each with results indicating a diminution in pain intensity in both clonazepam and pregabalin cohorts.

The dual administration approach was explored by Amos *et al*. [15], who documented a notable decline in pain intensity in patients administered with 0.5 mg of clonazepam orally thrice daily for six months. Similarly, Shin *et al*. [21] in a 6 weeks study, tested the efficacy of topical and systemic clonazepam on 41 BMS patients. Patients were instructed to take 0.75 mg clonazepam three times daily for the first 2 weeks (2.25 mg daily), with an increase of the dosage to 1.5 mg three times daily for the remaining 4 weeks (4.5 mg daily) if the patient didn’t report side effects or improve of the symptoms.

While these findings affirm the analgesic properties of clonazepam in BMS, the challenge of standardizing treatment due to the heterogeneity of administration routes and dosages remains. Additionally, a degree of uncertainty pervades the long-term efficacy of clonazepam, compounded by the potential for dependency associated with protracted systemic use. Clonazepam, used in the management of BMS-related symptoms, should be prescribed with caution due to the potential for dependency. Prescribing regulations vary across countries and often restrict its use to specific indications and specialist settings. Clinicians must therefore carefully consider both regulatory constraints and dependency risks. In some settings, topical formulations may offer a more practical alternative to systemic administration, serving as an adjunct for symptom control. The present evidence base, while supportive of clonazepam’s use in BMS symptom mitigation, necessitates further research to refine therapeutic protocols and ascertain optimal treatment outcomes for BMS patients.

### 3.2 Capsaicin

Capsaicin, a compound found in chili peppers, for its analgesic properties, has demonstrated efficacy in treating various peripheral neuropathies, in post-herpetic neuralgia, and in BMS [26].

Functioning as a Transient Receptor Potential Vanilloid 1 (TRPV1) receptor agonist localized on C fibers, capsaicin instigates neuronal activation accompanied by the release of pro-inflammatory agents, such as substance P, neurokinin A (NKA) and calcitonin-gene-related peptide (CGRP), leading to a phase of heightened sensitivity to pain [26]. Subsequent and continual administration results in a gradual desensitization of the TRPV1 receptor, rendering it less receptive to painful stimuli due to alterations in calcium ion influx, ultimately modifying receptor functions and neuronal architecture—thereby diminishing pain and burning sensations [27] (Fig. 4).

**Fig. 4.** Capsaicin and TRPV1 receptors. Capsaicin exerts its effects primarily through its interaction with TRPV1 receptors, which are localized in the plasma membrane of Aδ and C fiber primary afferents. The inactivation of voltage-gated Na^+^ channels and direct pharmacological desensitization of TRPV1 receptors in the plasma membrane may contribute to an immediate reduction in neuronal excitability and responsiveness. Prolonged or repeated exposure to capsaicin results in the desensitization of TRPV1 receptors. This desensitization reduces the sensitivity of sensory neurons to painful stimuli, leading to an analgesic (pain-relieving) effect. The exact mechanisms of desensitization also involve the depletion of neuropeptides like substance P, desensitization of the TRPV1 channels themselves, and modulation of downstream signaling pathways. TRPV1: transient receptor potential vanilloid subtype 1.

Notwithstanding its therapeutic potential in pain and inflammation management, its overactivation may induce cytotoxicity, which can damage cells and tissues [28].

Topical and systemic capsaicin applications for BMS have been examined.

Petruzzi *et al*. [29] investigated the effects of oral administration of capsaicin in patients with BMS. This pilot study involved administering 0.25% capsaicin capsules three times daily for 14 days and found significant decreases in VAS scores compared to placebo groups. Systemic capsaicin showed short-term efficacy for BMS but was associated with high gastric toxicity (32% *vs.* 0% in placebo), raising concerns about its long-term use. Further trials are needed to evaluate its safety and applicability [29].

Several studies have explored the topical administration of capsaicin in BMS patients using mouth rinse or oral gel.

Silvestre *et al*. [30] conducted a -blind, placebo-controlled study, which confirmed VAS score amelioration in subjects receiving a 0.02% capsaicin rinse for approximately 30 seconds, in 15 mL aliquots, thrice daily, when measured against a placebo. Similarly, in the study of Jørgensen, subjects were randomized to receive either 0.01% or 0.025% capsaicin oral gel applications on the tongue’s dorsal region, three times a day for a fortnight [31]. The study revealed a significant reduction in oral burning, with no notable efficacy disparity between the two gel concentrations. Moreover, Ricken *et al*. [32] corroborated these findings, with the 0.025% oral capsaicin gel formulation further evidencing its capacity to reduce VAS scores.

Additionally, Marino *et al*. [33] showed an improvement in symptoms using 250 mg of chilli powder emulsified in 50 mL of water with a dose concentration of 3.54 µg/mL of capsaicin in a group of BMS cohort. Azzi *et al*. [34] also contributed to the corpus of evidence, reporting a considerable decrease in oral discomfort among BMS patients subjected to a prolonged regimen of capsaicin mouth rinse applications.

Capsaicin may be a potential topical treatment for BMS, but prolonged or excessive use could cause mucosal damage, such as peeling or ulceration. Further research is needed to assess its efficacy and safety, determine the optimal concentration and application frequency, and investigate long-term outcomes to guide clinical practice.

### 3.3 Emerging and alternative topical therapies

Topical treatments studied within non-randomized controlled trial (RCT) contexts, including lidocaine, antihistamine agents, sucralfate, and lactoperoxidase oral solutions (Biotene®), have encountered disappointing efficacy [30, 35, 36]. Additionally, contemporary systematic analyses have revealed that neither benzydamine hydrochloride oral rinse (0.15%), topical urea (10%), nor chamomile extract (2%) do not significantly impact BMS symptomatology, with unsatisfactory outcomes [30, 35, 37].

The use of topical amitriptyline, which is commonly used to treat chronic neuropathic pain, was evaluated in a study by Lebel *et al*. [38] in which the authors compared the use of clonazepam drops with amitriptyline drops and observed a significant decrease in the VAS scores for both groups. The use of topical amitriptyline, which is commonly used to treat chronic neuropathic pain, was recently investigated in a retrospective real-world study by Lebel *et al*. [38]. In this study, 15 patients with BMS were treated with a topical solution (40 mg/mL), diluted by placing five drops (1 mg/drop) into 20 mL of water resulting in a 0.25 mg/mL solution administered twice daily. A significant reduction in pain intensity was observed with mild adverse events reported in 27% of cases, including somnolence, dry mouth, and dysgeusia. Although dry mouth was reported as a side effect in some patients, this may be advantageous in selected cases, particularly in those with sialorrhea. To reduce the incidence of dry mouth, strategies, such as further dilution of the solution, shortening mucosal contact time, or adjusting the frequency of application, may be considered.

Furthermore, in a more recent RCT by Hussein & El Marssafy (2025), patients used a mouthrinse prepared by dissolving a 10 mg or 25 mg amitriptyline tablet in 100 mL of distilled water. The rinse was applied for 2–3 minutes, three times daily for 8 weeks, resulting in dose-dependent pain reduction without any local or systemic adverse effects, further supporting the potential utility of topical amitriptyline in BMS [38, 39]. However, subjects treated with amitriptyline reported a worsening of pre-existing xerostomia, suggesting to avoid this formulation in BMS patients that report this additional symptoms.

Although these topical treatments have not demonstrated effectiveness when used independently, it is feasible to consider their use in conjunction with systemic medications, given that these treatments have not shown any side effects.

Recently, Gramacy and Villa assessed the effectiveness and safety of gabapentin (GB) topical solution (250 mg/mL) in a small retrospective study involving 19 BMS patients [40]. All patients were instructed to swish and spit 5 mL of the solution for 5 minutes, without swallowing, two to four times a day.

The hypothesis suggests that applying GB topically may block the alpha-2-delta1 (α2δ1) subunits, components of voltage-gated calcium channels (VGCCs) present in nociceptive neurons, and produce a local analgesic effect. GB could stabilize pain receptors in the mouth, potentially reducing pain [40]. Traditionally an antiepileptic, GB might also manage pain when used topically. However, further structured studies, including randomized and controlled trials with placebos, are needed to confirm these findings.

### 3.4 Antidepressants: efficacy and safety

Antidepressants (ADs), including tricyclics (TCAs), serotonin receptor antagonists and reuptake inhibitors (SARIs), selective serotonin reuptake inhibitors (SSRIs), and serotonin and noradrenaline reuptake inhibitors (SNRIs), have shown effectiveness in treating BMS. They offer pain relief and help with the accompanying psychological conditions. Studies indicate that improving mood disorders linked with BMS could indirectly lessen pain perception and bolster pain management strategies [41, 42]. While the exact mechanisms by which ADs alleviate pain are not completely understood, recent findings suggest their analgesic properties may function independently of their mood-enhancing effects [43, 44]. These drugs modulate pain transmission by elevating neurotransmitters like serotonin and noradrenaline levels in the synaptic cleft, which, in turn, may reduce the transmission of pain signals to the CNS by downregulating and desensitizing spinal dorsal horn receptors [45]. The initial delay in the effectiveness and subsequent tolerance to side effects might stem from the time required for receptor desensitization [46]. Moreover, higher neurotransmitter levels strengthen the descending inhibitory system, which is pivotal in managing nociceptive pathways that deliver pain signals [47] (Fig. 5).

**Fig. 5.** Descending pain modulation. There are several connections between the ascending and descending modulation systems. Descending modulation is mediated through projections from the amygdala to the periaqueductal gray (PAG), which also receives input from other brain areas including the hypothalamus. The PAG communicates with the rostral ventromedial medulla (RVM), which sends descending serotonergic projections to the Caudal Nucleus/Cervical Spinal Cord (NC/MS C1–C2), and with the locus coeruleus (LC), which sends inhibitory noradrenergic projections to the NC/MS C1–C2. Another descending modulation system involves neurons of the dorsal reticular nucleus (DRN). These circuits are sensitive to opioids because the PAG and the RVM are rich in µ receptors. The effect of serotonin (5HT) and noradrenaline (N) in the NC/MS C1–C2 can either inhibit or amplify pain, depending on the receptor subtype to which the neurotransmitters bind. Presynaptic activation of the 5-Hydroxytryptamine receptor 1B (5HT1B) receptor on the terminals of primary afferents has an anti-nociceptive effect because it reduces the release of glutamate in the NC/MS C1–C2, as does the activation of postsynaptic 5HT1A receptors on second-order neurons. In contrast, activation of the excitatory serotonin receptor 5HT7, identified in GABAergic interneurons of the NC/MS C1–C2, promotes the release of GABA, resulting in reduced excitability of secondary nociceptive neurons. Presynaptic activation of α2-adrenergic receptors inhibits the release of glutamate at the central nerve endings of primary nociceptive afferents and at the postsynaptic sites of second-order nociceptive neurons. SSRIs primarily act by blocking the reuptake of serotonin (5HT), increasing its availability in the synaptic cleft. In the diagram, they would modulate serotonergic neurons (blue pathways) at both the NC/MS C1–C2 and DRN, enhancing inhibitory control over pain pathways. SNRIs block the reuptake of both serotonin and norepinephrine, increasing their levels in the synapse. They would influence both serotonergic (blue pathways) and noradrenergic (red pathways) neurons, acting at NC/MS C1–C2 and LC to provide pain relief through enhanced inhibition and reduced excitation. TCAs inhibit the reuptake of serotonin and norepinephrine, similar to SNRIs, but also affect other neurotransmitters. Their action would be on both serotonergic and noradrenergic neurons, enhancing descending inhibitory pathways at NC/MS C1–C2 and LC, as well as potentially affecting other areas of pain modulation such as DRN. Vortioxetine has a multimodal mechanism: it inhibits serotonin reuptake, acts as an agonist on 5HT1A receptors, partial agonist on 5HT1B receptors, and antagonist on 5HT3, 5HT1D, and 5HT7 receptors. It would modulate serotonergic pathways (blue pathways) at various points, enhancing inhibitory signals and potentially reducing pain transmission through multiple receptor interactions. PAG: Mesencephalic periaqueductal gray; RVM: Rostral ventromedial medulla; NC/MS C1–C2: Caudal nucleus/Cervical spinal cord C1–C2; LC: Locus coeruleus; DRN: Dorsal reticular nucleus; µ-R: Opioid µ receptors; 5HT: Serotonin; N: Noradrenaline; α2: Noradrenergic receptor; TCAs: Tricyclic antidepressants; SSRI: Selective Serotonin Reuptake Inhibitors; SNRI: Serotonin and Noradrenaline Reuptake Inhibitors; GABA: gamma-aminobutyric acid.

Dysfunctions in these pathways can amplify chronic pain, underscoring the importance of serotonin and noradrenaline in the pain management process within BMS treatments [48].

Furthermore, ADs enhance synaptic neuroplasticity and mend disruptions in the hippocampus, amygdala, and cerebral prefrontal cortex connections by increasing Brain-Derived Neurotrophic Factor (BDNF) [49]. This aids in neuron restoration and contributes to chronic pain relief and the reduction of co-occurring anxiety and depression, particularly evident with long-term use [50].

Additionally, ADs provide pain relief by blocking sodium channels, thus preventing discharges in damaged nerves [51], and antagonizing N-methyl-D-aspartate (NMDA) receptors [52], implicated in the increased sensitivity characteristic of neuropathic pain [53].

Lastly, ADs may also offer anti-inflammatory benefits by decreasing pro-inflammatory cytokines such as Interleukin-6 (IL-6) [54], offering a potential therapeutic advantage in BMS treatment strategies where inflammation and elevated IL-6 levels are believed to play a critical role [55].

#### 3.4.1 Tricyclic antidepressants (TCAs)

TCAs, such as amitriptyline and nortriptyline, have demonstrated efficacy in managing BMS, albeit with varying degrees of effectiveness among individuals. Recent research by Goncalves *et al*. [37] reported a positive response rate of 74.3% in 35 BMS patients treated with amitriptyline [56]. The medication was administered orally at bedtime, with dosages ranging from 12.5 mg to 150 mg, and the treatment duration was six months. Interestingly, males exhibited a higher response rate (100%) to amitriptyline compared to females (67.9%).

Although the underlying mechanisms remain incompletely understood, possible explanations may involve sex-specific modulation of descending pain pathways or neurotransmitter activity. This citation strengthens the rationale for reporting such differences in our study. This aligns with recent findings by Nagamine *et al*. [57], which highlight sex-related differences in clinical responsiveness to amitriptyline in BMS. While the underlying mechanisms remain to be fully elucidated, they may involve sex-specific modulation of descending pain pathways and neurotransmitter systems. However, Nagamine evaluated the effectiveness of low doses of amitriptyline in 51 patients with BMS, who were divided into three groups receiving different dosages ranging from 5 to 30 mg [58]. The results indicated that the efficacy of amitriptyline for BMS was not dose-dependent, and the most effective dosage was between 10 and 15 mg.

Similarly, Kim *et al*. [59] observed a reduction in pain in 31 patients treated with nortriptyline at dosages ranging from 10 to 30 mg/day.

However, it’s crucial to consider that TCAs can be associated with several side effects, including dry mouth, constipation, blurred vision, urinary retention, dizziness, weight gain, sexual dysfunction, sedation, increased heart rate, and confusion or cognitive impairment [60]. These side effects may be more pronounced compared to those of SSRIs, SNRIs, and Vortioxetine (VO) [61]. Consequently, very gradual and cautious dose escalation is essential, even at low starting dosages, particularly in older adults, to enhance tolerability and minimize adverse effects.

Consequently, careful dose adjustments may be necessary, especially for older adults, to minimize these adverse effects [62]. Additionally, the risk of withdrawal symptoms with abrupt discontinuation of TCAs highlights the need for medical supervision when tapering off these medications.

In the past, TCAs were considered the primary treatment for BMS. However, given their broad range of side effects and the median age of BMS patients, who often present with multiple comorbidities, these agents should be prescribed cautiously. TCAs remain a valid therapeutic option, particularly when first-line treatments with better safety profiles are ineffective, but their use requires individualized dosing, progressive titration, and close clinical monitoring.

#### 3.4.2 Selective serotonin reuptake inhibitors (SSRIs) and serotonin and noradrenaline reuptake inhibitors (SNRIs)

SSRI and SNRI are being increasingly used in the treatment of BMS patients due to their effectiveness and milder adverse events compared with TCAs. When selecting an SSRI, factors such as side effect profiles and pharmacological properties, including drug half-life and interactions involving cytochrome P450 enzymes, are considered [63].

A review of multiple studies indicates that both paroxetine and sertraline have higher effectiveness rates compared to escitalopram and citalopram; fluoxetine has been evaluated in only one study.

In this context, Maina *et al*. [64] conducted an 8-week randomized trial with 70 BMS patients receiving either amisulpride (50 mg/day), paroxetine (20 mg/day), or sertraline (50 mg/day). All treatments were found to significantly alleviate BMS symptoms without severe adverse effects, that were similar across all groups.

Following this, a 12-week open-label, non-comparative prospective study by Yamazaki *et al*. [65] on 71 BMS patients reported a 70.4% remission rate of pain with paroxetine (10–30 mg/day), noting only minor and transient side effects. Another study by Ohga *et al*. [66] involving 43 BMS patients recommended initiating treatment with 10 mg/day of paroxetine, incrementally increasing it to 20 mg and 30 mg as needed, based on the patient’s response. This titration approach aimed to optimize therapeutic outcomes by minimizing adverse effects.

Finally, in a more extensive and long trial of 12 months, Adamo’s research included 150 BMS patients randomized to receive Vortioxetine (VO) (15 mg/day), paroxetine (20 mg/day), sertraline (50 mg/day), escitalopram (10 mg/day), or duloxetine (60 mg/day) [10]. The VO group exhibited the highest efficacy and shortest time to action with the fewest side effects. However, by the end of the study, all Ads were found to elicit a clinical response, with treatments being well-accepted and few dropouts. Among the SSRIs, sertraline was noted for better tolerability, a lower risk of drug interactions, and a minor impact on Corrected QT interval (QTc) prolongation—a significant consideration for patients with a history of myocardial infarction or other cardiac conditions—compared to paroxetine, which carried a higher risk of side effects, notably weight gain and sexual dysfunction [10].

Fluoxetine is approved for treating depression, but it also addresses anxiety disorders, bulimia nervosa, premature ejaculation, and recently, nociceptive pain [67]. Unlike other SSRIs, fluoxetine’s mechanism for alleviating pain may involve not only the serotoninergic system, but also the opioidergic system [68]. Although fluoxetine has a low affinity for opioid receptors, it appears to indirectly increase levels of opioid peptides, such as enkephalins and endorphins [69].

In a recent study on BMS, fluoxetine was evaluated in a placebo-controlled trial conducted by Zoric *et al*. [70]. The study involved 100 BMS patients, with half receiving fluoxetine and the other half a placebo. The treatment began with an initial dose of 20 mg/day for the first three months, which was subsequently maintained or increased to 40 mg/day for several additional months. Notably, 70% of patients treated with fluoxetine experienced significant reductions in Hamilton Depression and Anxiety (HAM-D and HAM-A), and VAS scores, demonstrating the drug’s effectiveness in treating mood disorders and alleviating pain. However, 20% of patients reported side effects, including transient nausea, occasional headaches, and dizziness.

Choosing SSRIs for treating BMS involves more than just their effectiveness in pain relief; it is crucial to consider their side effects and how they might impact patients. Each SSRI has unique characteristics that may make it more suitable for certain patients. For instance, paroxetine can cause significant withdrawal symptoms and has sedative effects, which might be either beneficial or detrimental. Fluoxetine, with its long half-life, minimizes withdrawal symptoms and is used for various conditions beyond depression, such as bulimia. Sertraline is often preferred for treating obsessive-compulsive disorder (OCD) due to its specific benefits in managing the condition.

A comprehensive evaluation of a patient’s mood and psychological conditions is crucial for selecting an SSRI that effectively treats BMS and supports overall mental health. Personalized treatment plans optimize both pain relief and psychological well-being in BMS patients.

Several studies have demonstrated the effectiveness of SNRIs, such as duloxetine, venlafaxine, and milnacipran, in treating various chronic pain conditions, including chronic low back pain, osteoarthritis, fibromyalgia, and peripheral diabetic neuropathy [43, 71].

Indeed, the increased level of noradrenaline caused by these drugs enhances the function of the periaqueductal grey, which is the origin of the descending pain inhibitory pathway, and the basal ganglia, which is the reward system involved in pain amelioration [72] (Fig. 6).

**Fig. 6.** Multifaceted mechanism of action of vortioxetine. Vortioxetine, administered at a dose of 10–20 mg, exerts a broad spectrum of effects through its interaction with various serotonin (5HT) receptors and the serotonin transporter (SERT). It acts as an agonist at 5HT1A receptors and a partial agonist at 5HT1B receptors, contributing to its antidepressant and pain control effects, respectively. Additionally, it inhibits serotonin reuptake by targeting SERT, leading to increased serotonin levels. Vortioxetine also functions as an antagonist at 5HT3, 5HT7, and 5HT1D receptors, which enhances its pro-cognitive effects and helps improve sleep. Furthermore, it promotes brain neuroplasticity by increasing brain-derived neurotrophic factor (BDNF) levels through interleukin-4 (IL-4) signaling.

In the study of Mignogna *et al*. [73] the authors found that 60 mg/day of duloxetine led to complete remission of symptoms in a 65-year-old woman with BMS. Similarly, Nagashima *et al*. [74] reported positive outcomes with duloxetine administered in a flexible dose ranging from 20 to 40 mg/day over a 12-week period. Another case report involved a 77-year-old female patient with BMS resistant to conventional treatments, who successfully managed her symptoms with duloxetine, thereby supporting its use in treatment-resistant BMS cases [75].

A study by Moon-Jong Kim and H. Kho found contrasting results. Four BMS patients treated with venlafaxine and nine with duloxetine (37.5–75 mg/day for venlafaxine, 30 mg/day for duloxetine) for four weeks showed limited relief after resistance to other treatments. While some patients improved, most did not experience significant symptom relief, and some discontinued due to intolerable side effects. This suggests that duloxetine’s efficacy may be limited in refractory BMS patients [76].

SNRIs affect both serotonin and norepinephrine and may cause side effects like increased blood pressure and heightened alertness, which can be either beneficial or detrimental. The choice between SSRIs and SNRIs for treating BMS depends on the patient’s specific symptoms and health conditions. SSRIs are preferred for patients sensitive to norepinephrine’s effects on heart rate and blood pressure, while SNRIs might be chosen for their energizing effects in patients with lethargic depression. The decision should be based on the individual’s health profile, disorder characteristics, and response to previous treatments, balancing efficacy and tolerability.

#### 3.4.3 Trazodone (SARI)

Trazodone has demonstrated efficacy in treating major depressive disorder. It has comparable antidepressant effects to other ADs, benefiting from its sleep-enhancing qualities and minimizing adverse effects on sleep continuity [77].

Trazodone exhibits dose-dependent pharmacologic effects. At higher doses (150–300 mg), it blocks serotonin transporters (SERT), thereby increasing serotonin levels to enhance mood. In contrast, lower doses (25–150 mg) primarily act as a sedative for insomnia by blocking histamine 1 (H1), 5-hydroxytryptamine 2A (5HT2A), and α1-adrenergic receptors [78].

Research into the use of trazodone for treating BMS remains inconclusive. An 8-week, placebo-controlled, double-blind trial by Tammiala-Salonen and Forssell examined the efficacy of 200 mg trazodone *vs.* placebo in 37 BMS patients. No significant improvement in pain relief was found for the trazodone group [79]. Choi *et al*. [80] reported that trazodone underperformed compared to other medications, such as GB, paroxetine, and clonazepam.

Low doses of trazodone improve sleep continuity and reduce sleep latency, effectively increasing total sleep time, as shown by studies [81]. This helps prevent early morning awakenings and complements SSRI or SNRI therapies for sleep disorders. While generally well-tolerated at low doses, trazodone can cause side effects like drowsiness and dizziness. Further research is needed to explore its broader potential and effectiveness in combination with other treatments.

#### 3.4.4 Vortioxetine (VO)

VO, a newer antidepressant, was approved in the USA in 2013 for treating Major Depressive Disorder (MDD) [82]. VO shares mechanisms of action with SSRIs while also having unique properties [83, 84].

VO is a distinctive AD that operates through a complex, multimodal mechanism involving multiple neurotransmitter systems (Fig. 6). Its primary action consists of inhibiting the SERT and consequently elevates the concentration of serotonin in the synaptic cleft. VO is the only drug that can modulate directly the serotonin (5HT) receptors activity, being a full agonist of 5HT1A, a partial agonist of 5HT1B, and an antagonist of the 5HT3, 5HT7 and 5HT1D receptors [85, 86].

VO’s binding affinity is dose-proportional. Experimental clinical studies have shown that VO engages, preferentially, SERT and 5HT3 at a lower dosage, between 5 and 10 mg, and engages all targets at a higher dosage of 20 mg.

VO acts as a full agonist at 5HT1A presynaptic receptors, accelerating desensitization and reducing the latency of action. It also stimulates postsynaptic 5HT1A receptors, increasing the release of neurotransmitters, like glutamate and noradrenaline. VO’s effects on 5HT1B receptors, combined with its 5HT1A receptor agonism, further regulate serotonin release by acting as a partial agonist at 5HT1B autoreceptors on presynaptic serotonergic neurons, leading to improved serotonergic transmission. This combined action contributes to VO’s overall antidepressant effect. Additionally, partial agonism at 5HT1B receptors increases the release of not only serotonin, but also glutamate, acetylcholine, histamine, and indirectly dopamine and noradrenaline [87].

Another unique property of VO is the increasing of activity of the glutamatergic pyramidal neurons in the prefrontal cortex and hippocampus through the antagonism of 5HT3 and a reduction of GABAergic transmission in a subset of inhibitory interneurons [88].

Additionally, the antagonism of 5HT7 increases the release of acetylcholine, a noradrenaline in the medial prefrontal cortex [85] directly involved in learning, memory, attention, and alertness [89].

Finally, the simultaneous action on 5HT3 and 5HT7 receptors regulates the production of pro-inflammatory cytokines, causing anti-inflammatory effects in the CNS [90]. VO increases the production of the cytokine IL-4, which helps control the immune response in the brain. This regulation supports the protective functions of astrocytes and encourages them to release BDNF, a beneficial brain protein. Additionally, it guides the transformation of microglia cells, part of the immune system in the brain, towards an anti-inflammatory state, thus reducing the creation of harmful inflammatory cytokines [88]. In addition, several studies have shown that VO can increase synaptic plasticity and promote a maturation of hippocampal granulated cell dendrites [91]. From an analysis of the literature, it is revealed that this brain neuroplasticity induced by the treatment with VO is greater than that induced by other Ads [88]. All these actions contribute to the pro-cognitive effect of VO [92].

Recently, VO has been demonstrated to improve sleep, through its agonism on 5HT1A and antagonisms on the other 5HT receptors. VO increases Rapid Eye Movement (REM) onset latency and decreases time spent in REM sleep; its favorable effects could be related to the blocking of the 5HT-3 receptors that may improve non-REM sleep and increase slow wave sleep, while the antagonism of 5HT-7 and 5HT-1D suppresses REM sleep [93].

Moreover, VO could have a role in the pain management of BMS. In a recent experimental study, VO has shown a strong analgesic activity compared with venlafaxine [94].

First, the blocking of SERT results in the up-regulation of biogenic amine neurotransmitters such as 5HT and noradrenaline in the synaptic cleft of the central and peripheral nervous system, which can modulate pain transmission. Secondly, the direct modulation of receptor activity contributes to the allodynic action of the drug [95]. Studies on rats have demonstrated that the antagonism of the 5HT3 receptors increases the noradrenaline levels in the hippocampus and that the agonism of the 5HT1A receptors increases the noradrenaline levels in the hypothalamus and hippocampus [96].

The rise in certain neurotransmitters, coupled with the decreased responsiveness and sensitivity of receptors in the spinal dorsal horns over time, helps lessen the pain signals traveling to the CNS. Furthermore, the simultaneous boost in the levels of 5HT, noradrenaline, and dopamine within the CNS enhances the effectiveness of the descending inhibitory system, which helps control the pathway for pain signals moving upward [93].

In the condition of neuropathic pain, the serotonergic pathway descending from the lower brainstem to the dorsal horn of the spinal cord is hyperalgesic and this effect is mediated by the activation of the 5HT3 receptors by 5HT. Conversely, the activation of the 5HT7 receptors has an analgesic activity [97].

Measurements of occupancy of the 5HT receptors in mice have shown that the saturation of the receptors by the drug is related to the dosage. At a dosage of 10 mg daily, VO saturates and blocks all the 5HT3 receptors but only 20% of 5HT7 receptors [98]. Presumably, at a dosage of 15 mg daily, no significant changes would be found. Therefore, at therapeutic dosage, VO blocks all 5HT3 receptors reducing hyperalgesia but leaving the majority of the 5HT-7 receptors free and subsequently preserving analgesia mediated by these receptors [94]. This is an interesting hypothesis which needs further investigation.

The complex mechanism of action of vortioxetine contributes to its antidepressant, anxiolytic, pro-cognitive, and analgesic properties, providing comprehensive relief from depression, anxiety, cognitive impairment, and pain [95].

No drug interactions or adverse effects, such as QTc interval prolongation, sexual dysfunction, or weight gain, were identified in several randomized controlled on MDD [99].

Adamo *et al*. [10, 87] conducted two clinical trials that demonstrated the effectiveness of VO in treating BMS. In the first trial, 30 patients received VO alongside topical clonazepam for 12 months, showing significant improvements in various health scores [87]. A larger, 12-month trial with 150 participants compared VO to other antidepressants like paroxetine and sertraline [10]. VO was found to have quicker antidepressant effects and better pain control, and was preferred by patients due to its cognitive benefits. VO also led to a high rate of clinical response and remission, with 96.6% of patients showing functional recovery at 6 months [100]. Side effects were minor and infrequent compared to other medications [10]. These results position VO as a leading treatment for BMS, significantly improving patients’ quality of life by reducing pain, anxiety, and depression, and enhancing cognition and sleep [101].

### 3.5 Antiepileptics (pregabalin and gabapentin)

Pregabalin (PGB) and gabapentin (GB) play a significant role in managing both acute and chronic pain by reducing pain intensity and opioid use, thereby improving quality of life through their modulation of pain pathways [102]. Both drugs work by binding to the alpha-2-delta subunit of voltage-gated calcium channels, thereby inhibiting calcium influx and reducing the release of neurotransmitters, including glutamate, noradrenaline, and substance P, involved in pain signaling [103] (Fig. 3).

PGB may also activate the descending noradrenergic system, which further alleviates neuropathic pain, and improves sleep quality and anxiety [104]. PGB is approved by the Food and Drug Administration (FDA) for neuropathic pain and for anxiety disorders [105].

Various studies have shown the effectiveness of PGB in treating BMS, especially when other treatments fail. Ito *et al*. [106] found PGB effective in five patients who did not respond to SNRI treatments. Heo *et al*. [107] reported that PGB provided pain relief for 70% of 19 BMS patients who were unresponsive to clonazepam. Choi *et al*. [80] also supported PGB’s benefits in a study of 33 BMS patients.

PGB is also considered for combined use with SSRIs or SNRIs in treating other chronic pain conditions and fibromyalgia when single-drug treatments are ineffective. Additionally, a study by Adamo with 203 patients showed that adding PGB (75 mg–150 mg/day) to VO (20 mg/day) increased its effectiveness. The study demonstrated that adding PGB to treatments with VO or SSRIs/SNRIs showed a positive response in the majority of patients, with the VO-PGB combination having a quicker onset and fewer side effects. This therapeutic strategy may be especially beneficial in cases where first-line BMS treatments have failed.

Research on the use of GB for treating BMS remains limited and yields mixed results. In a randomized, double-blind, controlled trial involving 120 BMS patients, Lopez-D’Alessandro *et al*. [108] found that administering alpha-lipoic acid (600 mg/day) and GB (300 mg/day) for two months provided greater pain relief compared to placebo or drug individually. This suggests that a combination of medications targeting different levels of the nociceptive system can be beneficial in managing this syndrome [108].

Despite these promising results, research on PGB’s use in BMS is still mixed. Some studies, like those by Heckmann *et al*. and White *et al*., found GB, a drug similar to PGB, did not significantly relieve BMS pain [109, 110]. Based on the currently available data, pregabalin may be preferred over gabapentin for BMS management, although further research is essential to confirm its effectiveness [111].

### 3.6 Antipsychotics

Antipsychotics, traditionally used for schizophrenia and depression, may also help manage chronic pain, including conditions like trigeminal neuralgia and diabetic neuropathy [112].

Specifically, second-generation antipsychotics, also known as atypical antipsychotics, impact various neurotransmitter systems in the brain. They block dopamine (D2) receptors and affect adrenergic, acetylcholine, catecholamine, histamine, and serotonin receptors. They also inhibit NMDA and AMPA receptors, which block glutamatergic transmission. In an animal model, this inhibition showed potential for pain modulation [52].

Given their broad effects on neurotransmitters, atypical antipsychotics have been considered for treating BMS, especially in patients who do not respond to ADs therapy.

The antipsychotics used in BMS include olanzapine, aripiprazole, quetiapine, levosulpiride, and amisulpride.

Ueda *et al*. [113] reported significant pain relief with olanzapine (2.5–5 mg/day) in two patients unresponsive to milnacipran and paroxetine.

A low dose of aripiprazole (1 mg/day) demonstrated efficacy in treating BMS in a 66-year-old woman resistant to other antidepressants [114]. Additionally, Takenoshita *et al*. [115] reported that two patients unresponsive to amitriptyline alone experienced substantial pain reduction when a low dose of aripiprazole was added to their regimen. These positive effects were maintained for over two years.

Poyurovsky *et al*. [116] reported the effectiveness of quetiapine fumarate (50 mg/day) in a 50-year-old woman suffering from both BMS and OCD. She had previously undergone treatment with escitalopram, clonazepam, and fluoxetine, none of which provided relief. However, after just one week of starting quetiapine, she experienced rapid improvement in both depressive and BMS symptoms. Notably, there was no recurrence in the following three years of observation.

An 8-week single-blind study compared amisulpride (50 mg/day) with SSRIs in treating BMS. All treatments improved BMS symptoms significantly, and amisulpride had a shorter response latency than SSRIs, making it potentially more suitable for initial therapy [64]. Another study found significant improvement in BMS symptoms over 24 weeks with amisulpride (50 mg/day). The treatment was well tolerated with no serious adverse effects.

Finally, a study using levosulpiride (100 mg/day for 8 weeks) found partial improvement in 28 out of 39 patients. However, no complete remission was reported, and the response was more notable in patients with shorter disease durations [117].

Despite these encouraging findings, antipsychotic safety profiles are a concern, particularly for elderly patients. Adverse effects like QT prolongation, metabolic syndrome, extrapyramidal symptoms, weight gain, and sedation can be significant challenges [58]. Physicians should titrate doses carefully and monitor patients to minimize these risks while maximizing therapeutic benefits.

A careful evaluation of risks and benefits is necessary before prescribing antipsychotics for BMS, as most of the available evidence derives from case reports rather than controlled trials. These medications may, however, offer therapeutic value in selected patients with comorbid psychiatric conditions, such as OCD. Combination therapy involving low-dose antipsychotics alongside SSRIs/SNRIs or antiepileptics has shown preliminary promise, but further studies are needed to define optimal regimens and dosing strategies. Ultimately, larger-scale clinical trials are essential to determine the role of antipsychotics in BMS management. Until such data are available, clinicians should proceed with caution, particularly when considering these treatments in routine practice.

## 4. Dietary supplements

### 4.1 Melatonin

Research indicates that melatonin has potential therapeutic benefits for managing chronic pain. Yang *et al*. [118] reported that melatonin reduces myocardial susceptibility to chronic pain-related stress by inhibiting necroptosis and minimizing oxidative stress in rodent models. Additionally, Kaur and Shyu highlighted melatonin’s neuroprotective properties, suggesting that chronotherapy could be a promising approach to chronic pain management, particularly for sleep-related discomfort [119]. Danilov and Kurganova found that melatonin helps normalize circadian rhythms, thereby improving sleep quality in patients with fibromyalgia, irritable bowel syndrome, and other conditions. Melatonin also provides analgesia via its receptors [120].

However, research on melatonin’s efficacy in treating BMS has yielded mixed results. A triple-blind, randomized clinical trial found that melatonin (12 mg/day) did not outperform placebo in alleviating BMS pain [121]. However, it improved anxiety scores and slightly increased sleep duration, while both placebo and melatonin had safe pharmacologic profiles. In contrast, a placebo-controlled trial by Nosratzehi *et al*. [122], using the same melatonin dosage, showed a reduction in burning sensations but no significant improvement in sleep quality.

In another prospective, double-blind study, a lower dose of melatonin (1 mg/day) effectively reduced burning sensations and improved the quality of life in BMS patients. Clonazepam showed similar results [123].

Given melatonin’s multifaceted properties in pain management, particularly its analgesic and neuroprotective effects, further exploration in clinical trials is warranted [124]. Despite mixed findings, its potential benefits in alleviating pain and improving mental health could offer a promising alternative or complementary approach for individuals with chronic pain conditions, including BMS.

### 4.2 Alpha lipoic acid (ALA)

ALA is sometimes used as a supplement in chronic pain management due to its potential anti-inflammatory and antioxidant properties [125]. Research suggests that ALA (600–800 mg daily) can improve nerve function by neutralizing free radicals and reducing oxidative stress, both of which can impact chronic pain conditions [126]. Diabetic peripheral neuropathy, fibromyalgia, certain musculoskeletal disorders, and BMS may benefit from ALA supplementation [127, 128]. However, studies on ALA’s efficacy in managing BMS have shown mixed results.

In a double-blind, placebo-controlled trial, 64% of patients receiving ALA showed improvement, with 69% maintaining the benefit one month post-treatment [129]. Additionally, López-D’alessandro and Escovich reported that combining ALA with gabapentin significantly reduced pain in BMS [108]. Furthermore, Femiano *et al*. [130] found a positive outcome when ALA was combined with psychotherapy.

Conversely, other studies found no significant difference between ALA and placebo in alleviating BMS symptoms. For instance, a randomized, double-blind study by Cavalcanti and da Silveira indicated that while some patients reported improvement with ALA, the results were not significantly different from placebo [131].

In summary, while ALA has shown potential benefits for some patients with BMS, results are not universally consistent, and further research is needed to establish clear guidelines for its use in this condition.

### 4.3 Palmitoylethanolamide (PEA)

PEA, a bioactive lipid related to endocannabinoids, is found throughout the body, including the brain. It is produced as a protective response to tissue damage and has anti-inflammatory, pain-relieving, and other therapeutic effects [132]. PEA works in pain management by activating a receptor called Peroxisome Proliferator-Activated Receptor Alpha (PPAR-α), which reduces inflammation by lowering the levels of certain inflammatory molecules [133]. It also competes with similar compounds to inhibit an enzyme called Fatty Acid Amide Hydrolase (FAAH), increasing levels of protective bioactive lipids and reducing inflammation [134].

Additionally, PEA affects TRPV1 receptor channels, which are important for managing neuropathic pain and inflammation, thereby helping to reduce pain perception [135]. By inhibiting FAAH, PEA indirectly increases levels of anandamide, a naturally occurring cannabinoid, enhancing its pain-relieving effects through what’s known as the “entourage effect” [136].

Research, including studies on its ultra-micronized form, has shown that PEA can significantly reduce symptoms in conditions like BMS [133]. A double-blind, randomized, placebo-controlled trial involving 35 BMS patients found that those treated with ultramicronized-PEA 600 mg micro-granules/day (Normast®, 935939708, Epitech Group SpA, Padua, Italy) experienced a significant reduction in burning sensation compared to the placebo group [137]. Moreover, it has been demonstrated that PEA, especially when combined with medications like gabapentin, effectively reduces pain in BMS patients [138]. However, more research is needed to fully understand how PEA works and to confirm its effectiveness in treating BMS either alone or combined with other treatments.

## 5. Non-pharmacotherapeutic approaches

### 5.1 Cognitive-behavioral therapy (CBT)

CBT is a non-invasive method used to manage chronic pain by addressing the psychological and emotional aspects that can intensify pain perception [139]. CBT operates on the idea that thoughts, behaviors, and emotions are interconnected, and modifying these can alleviate undue emotional responses to pain [140].

CBT assists patients in recognizing and challenging negative thought patterns, such as catastrophizing, and encourages replacing them with more positive and balanced thoughts, which can lessen distress and enhance pain tolerance [141]. It promotes patient motivation and independence, encouraging participation in enjoyable activities to break the cycle of pain and depression [141].

The therapy includes relaxation techniques, like deep breathing, progressive muscle relaxation, and guided imagery, to reduce stress and muscle tension, which can amplify pain [142].

Additionally, CBT provides coping strategies, such as problem-solving and seeking social support, which aid in managing the emotional impacts of pain [140]. Biofeedback is used to help patients monitor and respond to physical signs of stress, such as muscle tension and heart rate, allowing them to apply relaxation techniques effectively to decrease pain intensity [143].

CBT is considered an effective intervention for managing BMS symptoms especially when combined with medications or other treatments as demonstrated by studies and a systematic review [144] (Table 2).

**Table 2.** Non-pharmacotherapeutic approaches in burning mouth syndrome (BMS).

Aspect		Description	Positive Effects/Benefits
Cognitive-behavioral therapy (CBT)			
	Cognitive Restructuring	Identification and modification of maladaptive thoughts, replacing them with adaptive and positive alternatives	Reduced catastrophizing, promotion of a healthier perspective on pain
	Behavioral Activation	Encourages engagement in pleasurable and mood-enhancing activities	Mitigation of depressive symptoms, improved motivation, and reduction in pain perception
	Exposure Therapy	Gradual exposure to oral triggers causing discomfort to reduce avoidance behaviors	Desensitization to painful stimuli, decreased oral pain intensity
	Relaxation Techniques	Incorporation of progressive muscle relaxation and mindfulness meditation	Reduction of stress, muscle tension, and overall pain perception
	Biofeedback	Monitoring of physiological responses to stress and pain, providing real-time feedback to patients	Enhanced awareness of pain triggers, improved coping strategies through visualization and relaxation
	Problem-Solving Skills	Development of practical problem-solving approaches to manage everyday challenges	Increased sense of control over pain and reduced psychological distress
	Support Network Building	Encouragement to seek supportive social interactions and reduce isolation	Improved emotional well-being, establishment of stronger social connections
Others Non-Pharmacotherapeutic Approaches			
	Low-level laser therapy (LLLT)	Non-invasive therapy using near-infrared light (600–1100 nm) to reduce inflammation and pain via peripheral nerve modulation	Significant pain relief, reduced pro-inflammatory cytokines, well tolerated, high patient acceptance, potential improvement in quality of life
	Transcranial Magnetic Stimulation (TMS)	Non-invasive brain stimulation (rTMS/Transcranial Direct Current Stimulation (tDCS)) that modulates cortical activity through magnetic or direct electrical currents	Effective in reducing pain intensity, especially in treatment-resistant BMS; potential cognitive and emotional benefits; generally safe and well tolerated
	Lifestyle Optimization	Adoption of healthy habits (diet, exercise, sleep, stress reduction) to counteract brain frailty and systemic risk factors	Improved pain control, enhanced cognitive function, reduced inflammation, better quality of life, support for pharmacological therapy, healthier aging

Individual and group CBT intervention (1–2 sessions) treatment has been shown to reduce both pain and anxiety levels in patients [145, 146].

Although the treatment has considerable and lasting effects, which can last up to 12 months, a full course of CBT poses financial challenges due to the high treatment costs [147].

Streamlining the CBT protocol to focus exclusively on psychoeducation can significantly reduce costs [148]. This strategy involves offering patients comprehensive information about BMS, including its characteristics, underlying mechanisms, and available treatment options, such as medications [149]. Providing education helps alleviate patients’ concerns about the potential severity of the condition. Emphasizing the importance of maintaining a normal lifestyle despite fluctuating symptoms also leads to improved patient outcomes [150].

### 5.2 Low-level laser therapy (LLLT)

LLLT, also known as photo biomodulation therapy, is a non-invasive, chair-side treatment that uses near-infrared light to provide analgesic and anti-inflammatory effects [151]. It is a promising therapy for reducing pain in BMS and may positively influence quality of life and mental health. The wavelengths typically range from 600 to 1100 nm, offering an optimal window for tissue penetration [152, 153]. This range promotes peripheral nerve regeneration and reduces pro-inflammatory cytokines like IL-6 and Tumor Necrosis Factor-alpha (TNF-α) [154]. The analgesic and anti-inflammatory effects prevent the depolarization of peripheral C fibers by reducing action potential amplitude and slowing impulse conductivity velocity [155].

Several studies emphasize LLLT’s efficacy over placebo and alternative treatments such as clonazepam, particularly when lower treatment frequencies (1–2 times weekly) are employed over extended periods. In a recent systematic review and meta-analysis by Lu *et al*. [156], the authors analyzed 14 RCTs involving 550 patients. They found that LLLT significantly reduced burning pain when administered at frequencies of ≤2 times per week, with treatment durations longer than four weeks yielding better outcomes. While the effects on quality of life and negative emotions were positive but non-significant, LLLT was well tolerated without serious adverse effects and garnered high acceptance among BMS patients [156].

The positive biological effects and absence of severe adverse outcomes make LLLT a compelling option for managing BMS [157]. However, comparisons between studies are hindered by inconsistencies in irradiation parameters, such as power, fluence, exposure time, and continuous versus pulsed emission, and no standardized treatment protocol currently exists. Further research is needed to establish optimal parameters and determine overall efficacy [156].

### 5.3 Transcranial magnetic stimulation (TMS)

TMS techniques, particularly repetitive transcranial magnetic stimulation (rTMS) and transcranial direct current stimulation (tDCS), have shown promising results in managing BMS, especially when other treatments are ineffective [158, 159]. rTMS is a non-invasive brain stimulation technique that employs a coil to generate brief magnetic pulses, which penetrate the scalp and skull to induce electric currents in specific brain regions [160]. The treatment is typically administered daily over several weeks [159]. It is approved for treating MDD resistant to conventional treatments and is being explored for other neuropsychiatric conditions, such as anxiety disorders, OCD, and Post Traumatic Stress Disorder (PTSD) [161]. tDCS, on the other hand, is a non-invasive neuromodulation technique that applies weak direct electrical currents to the scalp via electrodes [162]. It aims to modulate cortical excitability by delivering anodal (excitatory) or cathodal (inhibitory) stimulation to specific brain regions [163]. Although not yet universally approved for clinical use, research indicates its potential for treating various neurological and psychiatric conditions, such as depression, chronic pain, and cognitive rehabilitation post-stroke [162].

A randomized controlled, single-blind study demonstrated that daily rTMS sessions over the left prefrontal cortex significantly reduced pain intensity in 20 BMS patients who received a total of 30,000 pulses at 10 Hz [159]. Approximately 75% of patients reported over 50% pain reduction following the treatment [158]. A pilot case study involving a 74-year-old female with BMS for two years showed that 10 tDCS sessions (using a combined protocol with anodal stimulation of the left dorsolateral prefrontal cortex) paired with exercise and cognitive training resulted in reduced pain perception and improved quality of life, with the benefits correlating with improved cognitive function, although pain relief was temporary [160].

TMS is generally well tolerated, although mild and transient adverse effects, such as headache, scalp discomfort, or fatigue, have been reported; rare but serious events like seizures are extremely uncommon and typically associated with predisposing conditions [160, 163]. TMS techniques offer viable alternatives especially in BMS patients resistant to conventional treatment for reducing pain intensity. Additional research efforts are needed to minimize bias, improve quality, and identify optimal brain stimulation parameters to enhance their efficacy.

## 6. Lifestyle optimization in BMS

Recent research suggests that BMS might be an early sign of brain frailty, which could speed up brain aging and raise the risk of neurodegenerative diseases [59, 164] (Figs. 1,2). Unhealthy lifestyle habits such as smoking, lack of exercise, and obesity, along with cardiovascular risks and other health issues, may contribute to premature brain aging, increasing chronic pain and cognitive problems [165]. Conditions like high blood pressure and high cholesterol can also lead to brain issues that worsen pain and cognitive function [166]. Recent evidence indicates that hypertension is significantly more prevalent in BMS patients compared to controls, with age, comorbidities, drug consumption, and anxiety emerging as potential predictors of this association. For this reason, prevention through early detection and management of modifiable risk factors represents a key strategy in the comprehensive care of BMS patients [167].

To manage chronic pain like BMS, current strategies emphasize adopting healthier lifestyle habits [41]. This includes eating a diet rich in antioxidants, staying hydrated, and ensuring adequate intake of vitamins like B12 and folate for nerve and cognitive health. Regular physical activity, including aerobic and strength exercises, and stress-reducing practices like yoga or tai chi, are encouraged to boost brain health and reduce cognitive decline [168].

Improving sleep hygiene, such as sticking to a regular sleep schedule and cutting down screen time before bed, is essential for brain health. Quitting smoking also significantly reduces health risks related to oxidative stress and inflammation [169].

Given the frequent co-occurrence of BMS with systemic conditions in elderly patients, and the syndrome’s clinical heterogeneity, lifestyle optimization, including patient education, stress and anxiety management, and supportive non-pharmacological strategies, plays a crucial role in improving quality of life and supporting pharmacological interventions [41].

Making these lifestyle changes can greatly improve oral and cognitive health, slow the progression of BMS, and enhance overall well-being [42]. A comprehensive plan that includes these modifications can help manage BMS effectively and promote healthier aging of the brain [170].

Although many of these lifestyle interventions are shared with other chronic conditions, their application in BMS is increasingly supported by research on nociplastic and neuropathic pain. Evidence from related disorders, such as fibromyalgia and temporomandibular disorders shows that addressing modifiable risk factors, enhancing neuroplasticity, and managing stress and anxiety can meaningfully contribute to improved outcomes in BMS patients [171, 172].

## 7. Limitations

This article is a narrative review. As such, it may be subject to selection bias and does not follow the standardized protocols of systematic reviews. The literature was selected through targeted searches of PubMed, Scopus, and Web of Science, using combinations of relevant keywords such as Burning Mouth Syndrome, neuropathic pain, treatment, topical therapies, antidepressants, telemedicine, and brain aging. Articles were included based on their relevance to the clinical, pathophysiological, and therapeutic aspects of BMS, with a focus on original studies, systematic reviews, and guidelines in English. Reference lists of selected articles were also screened manually.

While this approach allowed for flexibility in addressing emerging and interdisciplinary themes, it also implies that some studies may have been inadvertently excluded. Therefore, the conclusions presented should be interpreted as an expert-informed synthesis rather than a fully comprehensive or reproducible evidence base.

## 8. Conclusions and future direction

While BMS has well-established diagnostic criteria and can often be identified through careful evaluation of patient-reported symptoms and the absence of mucosal lesions, its recognition in clinical practice remains suboptimal due to limited awareness and insufficient training among general healthcare providers. This, combined with an incomplete understanding of its etiopathogenesis and disease progression, makes it challenging to tailor treatment strategies, highlighting the need for personalized therapeutic approaches to optimize patient care and reduce frustration.

Comprehensive strategies encompassing pharmacological, non-pharmacological, and lifestyle interventions are crucial for achieving full functional recovery. Considering BMS’ potential as an early marker for neurodegenerative diseases, healthcare professionals should prioritize lifestyle optimization in tandem with treatment.

Emerging digital resources and telemedicine platforms have the potential to significantly enhance patient care, offering real-time monitoring, education, and access to multidisciplinary teams while reducing geographic and logistical barriers.

Telehealth can deliver personalized psychoeducation, cognitive-behavioral therapy, and adherence support, while tracking symptoms and treatment efficacy through digital tools. However, for chronic pain patients, particularly older adults with BMS, these tools must complement, rather than replace, the essential human connection with clinicians, given potential barriers to digital access and the importance of empathetic, in-person care. When thoughtfully integrated, these technologies can support a more accessible and efficient model of care, helping improve patient engagement and quality of life [170].
